# Basic and Clinical Research in Wound Healing

**DOI:** 10.3390/biomedicines11051380

**Published:** 2023-05-06

**Authors:** Gerrit Grieb

**Affiliations:** 1 Department of Plastic Surgery and Hand Surgery, Gemeinschaftskrankenhaus Havelhoehe, Kladower Damm 221, 14089 Berlin, Germany; gerritgrieb@gmx.de; 2 Department of Plastic Surgery and Hand Surgery, Burn Center, Medical Faculty, RWTH Aachen University, Pauwelsstrasse 30, 52074 Aachen, Germany

Wound healing in general is a complex physiological process. There are four phases of wound healing: the hemostatic phase, the inflammatory phase, the proliferative phase—including formation of granulation tissue, re-epithelialization, and matrix formation subphases—and the remodeling phase [1]. The timing of these phases must be appropriate for the wound to heal. However, due to different underlying local conditions such as hypoxia, vasoconstriction, or infection, and systemic diseases as atherosclerosis, diabetes, cardiovascular diseases, and immunodepression including medications such as glucocorticoids or chemotherapy, wound healing can be disturbed during one or more of these phases. Thus, proper treatment of wound healing disorders is very complex because several potential pathological factors must be considered to identify the proper target to be treated. This extensive process can result in high patient morbidity and wound care costs. Indeed, the annual cost of managing wounds is estimated to be $25 billion in the United States alone [2].

Many attempts have been made to study wound healing and wound healing disorders. In vitro investigations to evaluate what happens at the molecular level are important to sufficiently understand the underlying physiological and pathophysiological processes. Single or multiple factors involved in local inflammation, perfusion, and hypoxia are of particular interest. In addition to these in vitro investigations, the field of tissue engineering has emerged. Tissue engineering with respect to wound healing represents the in vitro engineering of different skin components or tissues and has become a promising field to treat abnormal wound healing [3]. Complex in vitro–engineered tissue that incorporates different biomaterials such as collagen or fibrin combined with different cell types including keratinocytes, fibroblasts, adipocytes, endothelial cells, and mesenchymal stem cells has presented promising results for potential clinical applications. Frequently, methods or models established in vitro can be successfully transferred to in vivo experiments to further clarify the physiological and pathophysiological processes of wound healing.

Finally, clinical investigations that evaluate potential products for local wound application are essential to assess the potential clinical use of different wound dressings. Multiple artificially produced or modified wound dressings based on materials such as collagen, hydrogels, or hydrocolloids are very promising. These clinical products are often combined with anti-infective substances such as silver or polyhexanide to treat infected wounds. Natural products such as alginates, birchbark, and manuka honey have also been widely investigated [4,5]. Despite the promising results, more randomized controlled trials, especially on the treatment of wound healing disorders or secondary wound healing, are essential to address the lack of evidence.

This Special Issue addresses a broad spectrum of research in wound healing, extending from in vitro and in vivo experiments to clinical studies and therapeutic applications. These aspects should be of great interest to the readers of Biomedicines.
